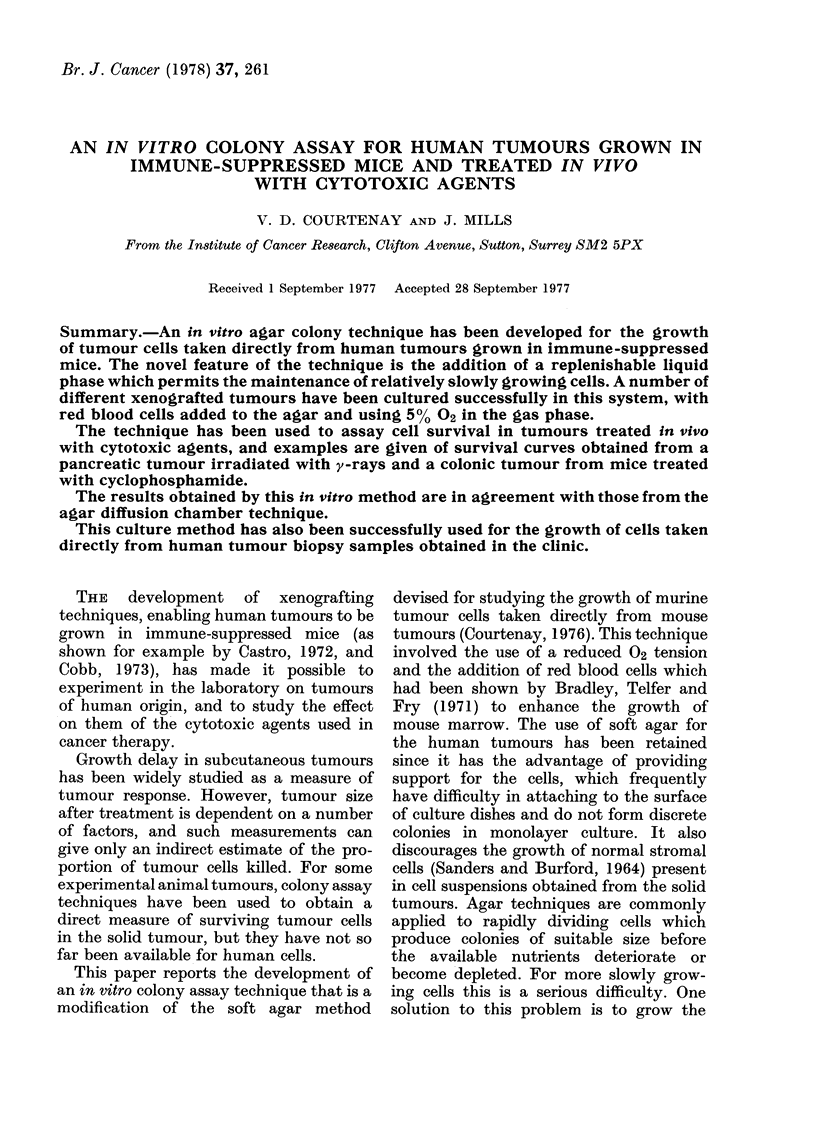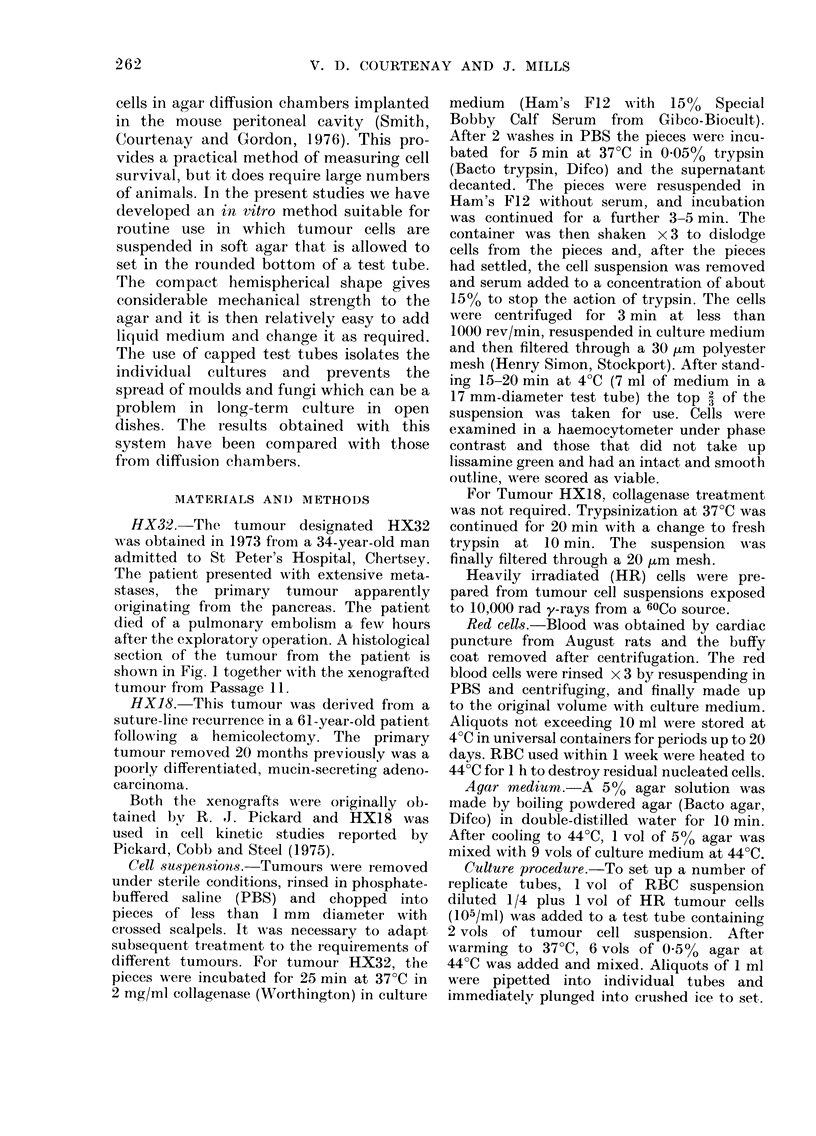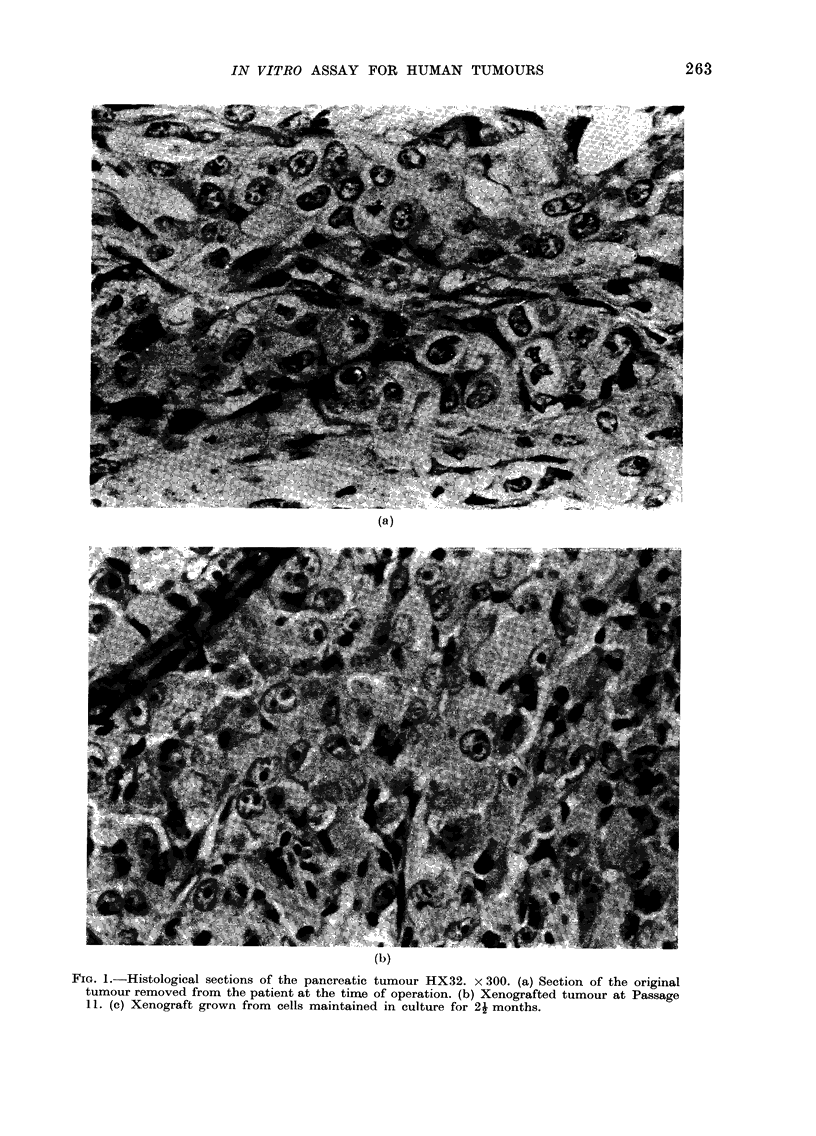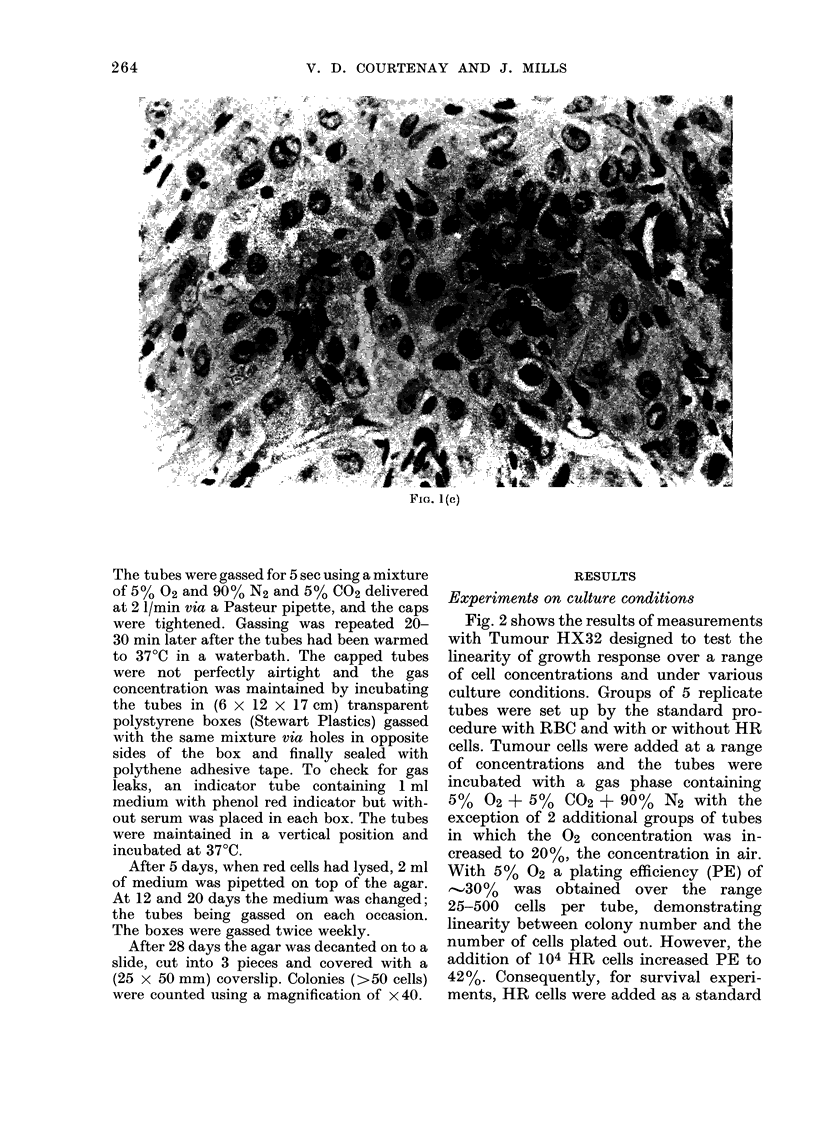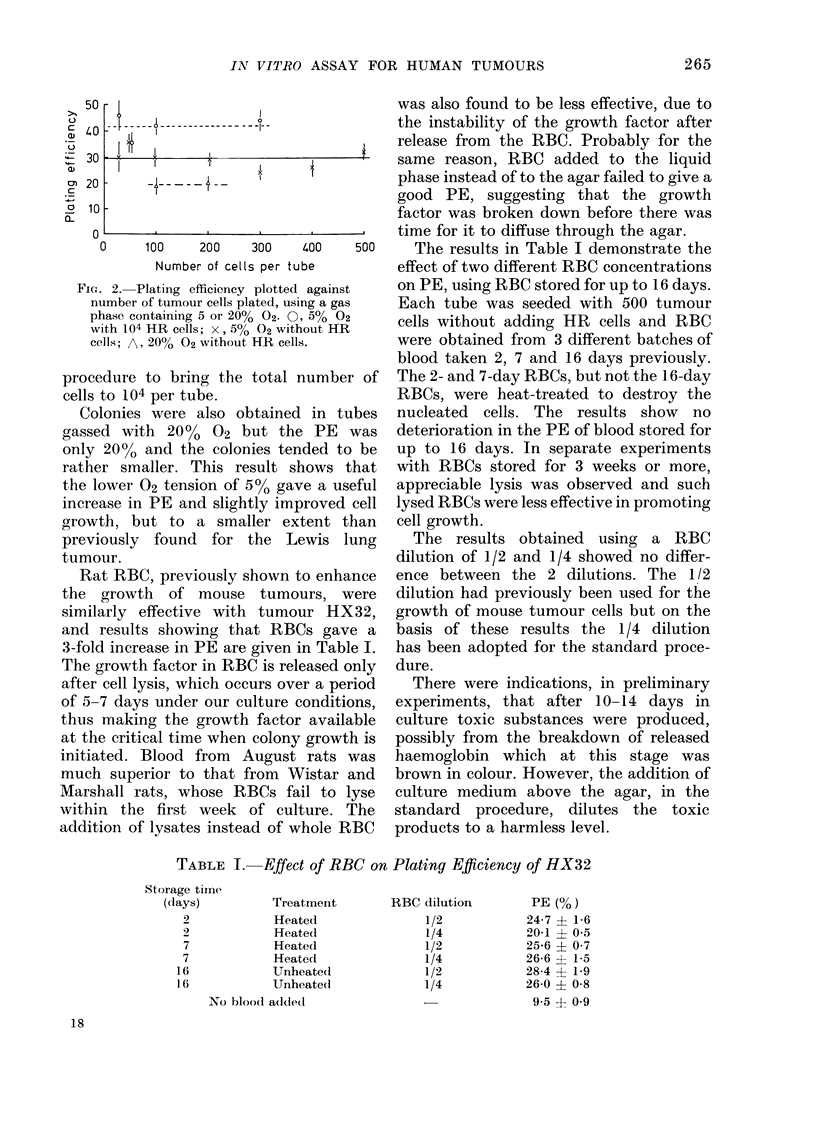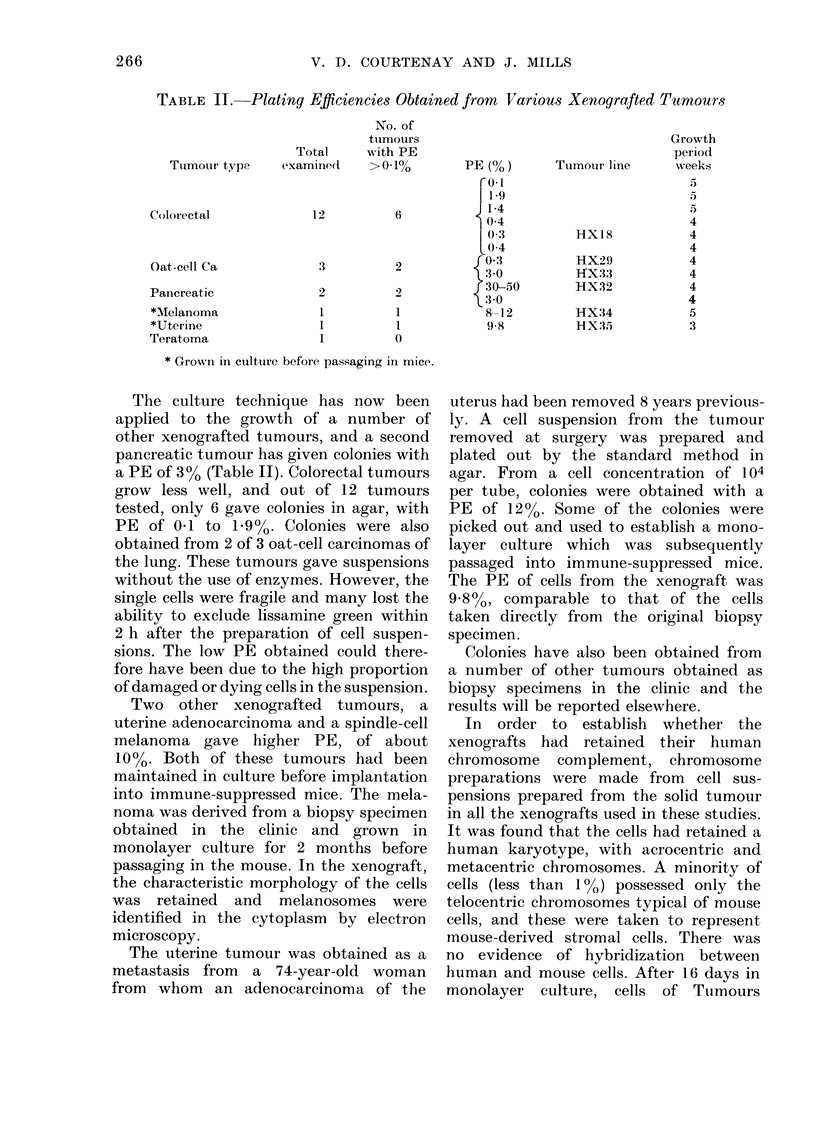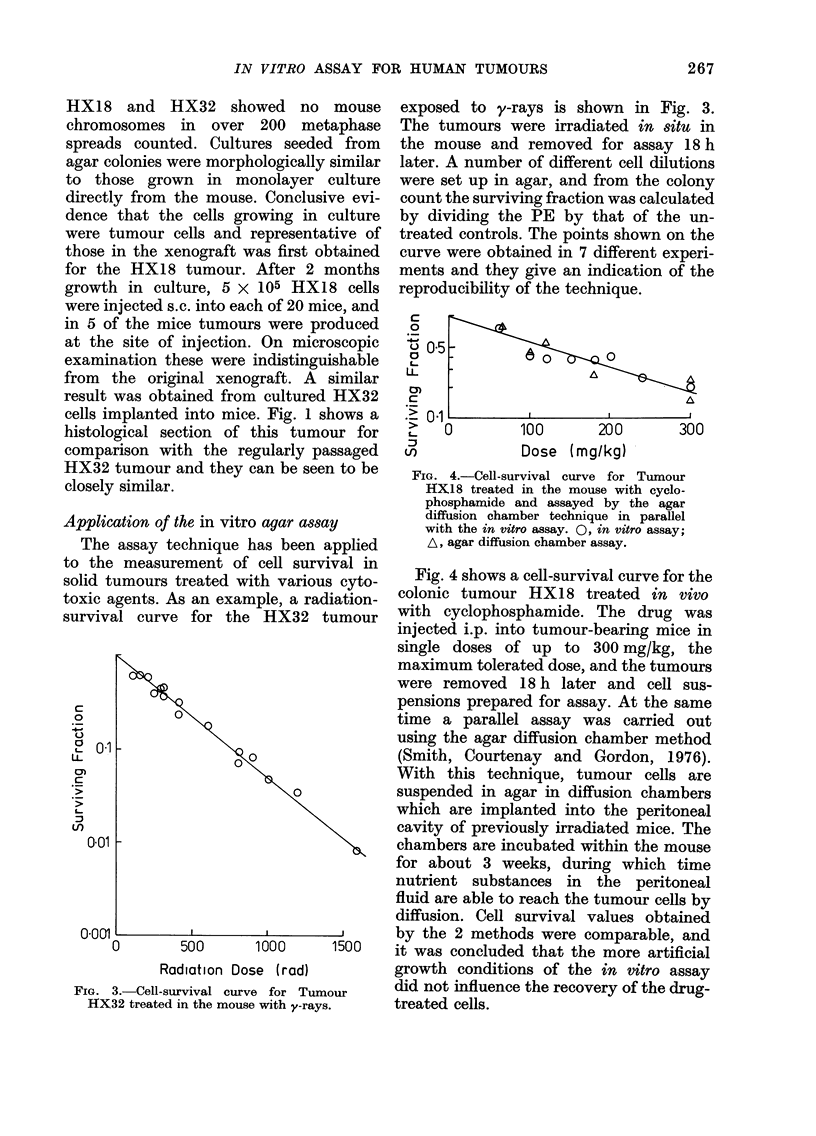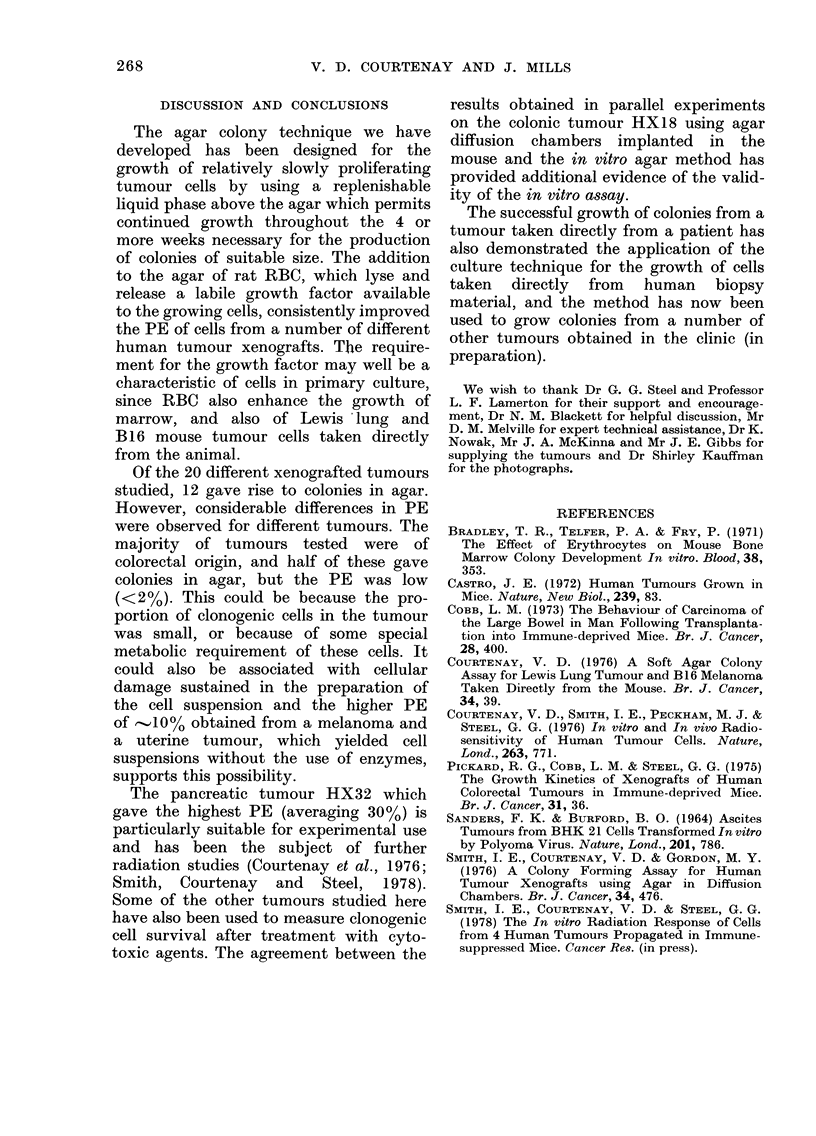# An in vitro colony assay for human tumours grown in immune-suppressed mice and treated in vivo with cytotoxic agents.

**DOI:** 10.1038/bjc.1978.35

**Published:** 1978-02

**Authors:** V. D. Courtenay, J. Mills

## Abstract

**Images:**


Br. J. Cancer (1978) 37, 261

### AN *IN VITRO* COLONY ASSAY FOR HUMAN TUMOURS GROWN IN IMMUNE-SUPPRESSED MICE AND TREATED *IN VIVO* WITH CYTOTOXIC AGENTS

V. D. COURTENAY AND J. MILLS

*From the Institute of Cancer Research, Clifton Avenue, Sutton, Surrey SM2 5PX*

Received 1 September 1977 Accepted 28 September 1977

**Summary.—An *in vitro* agar colony technique has been developed for the growth of tumour cells taken directly from human tumours grown in immune-suppressed mice. The novel feature of the technique is the addition of a replenishable liquid phase which permits the maintenance of relatively slowly growing cells. A number of different xenografted tumours have been cultured successfully in this system, with red blood cells added to the agar and using 5% $O_2$ in the gas phase.**

**The technique has been used to assay cell survival in tumours treated *in vivo* with cytotoxic agents, and examples are given of survival curves obtained from a pancreatic tumour irradiated with $\gamma$-rays and a colonic tumour from mice treated with cyclophosphamide.**

**The results obtained by this *in vitro* method are in agreement with those from the agar diffusion chamber technique.**

**This culture method has also been successfully used for the growth of cells taken directly from human tumour biopsy samples obtained in the clinic.**

THE development of xenografting techniques, enabling human tumours to be grown in immune-suppressed mice (as shown for example by Castro, 1972, and Cobb, 1973), has made it possible to experiment in the laboratory on tumours of human origin, and to study the effect on them of the cytotoxic agents used in cancer therapy.

Growth delay in subcutaneous tumours has been widely studied as a measure of tumour response. However, tumour size after treatment is dependent on a number of factors, and such measurements can give only an indirect estimate of the proportion of tumour cells killed. For some experimental animal tumours, colony assay techniques have been used to obtain a direct measure of surviving tumour cells in the solid tumour, but they have not so far been available for human cells.

This paper reports the development of an *in vitro* colony assay technique that is a modification of the soft agar method devised for studying the growth of murine tumour cells taken directly from mouse tumours (Courtenay, 1976). This technique involved the use of a reduced $O_2$ tension and the addition of red blood cells which had been shown by Bradley, Telfer and Fry (1971) to enhance the growth of mouse marrow. The use of soft agar for the human tumours has been retained since it has the advantage of providing support for the cells, which frequently have difficulty in attaching to the surface of culture dishes and do not form discrete colonies in monolayer culture. It also discourages the growth of normal stromal cells (Sanders and Burford, 1964) present in cell suspensions obtained from the solid tumours. Agar techniques are commonly applied to rapidly dividing cells which produce colonies of suitable size before the available nutrients deteriorate or become depleted. For more slowly growing cells this is a serious difficulty. One solution to this problem is to grow the cells in agar diffusion chambers implanted in the mouse peritoneal cavity (Smith, Courtenay and Gordon, 1976). This provides a practical method of measuring cell survival, but it does require large numbers of animals. In the present studies we have developed an *in vitro* method suitable for routine use in which tumour cells are suspended in soft agar that is allowed to set in the rounded bottom of a test tube. The compact hemispherical shape gives considerable mechanical strength to the agar and it is then relatively easy to add liquid medium and change it as required. The use of capped test tubes isolates the individual cultures and prevents the spread of moulds and fungi which can be a problem in long-term culture in open dishes. The results obtained with this system have been compared with those from diffusion chambers.

### MATERIALS AND METHODS

*HX32.*—The tumour designated HX32 was obtained in 1973 from a 34-year-old man admitted to St Peter's Hospital, Chertsey. The patient presented with extensive metastases, the primary tumour apparently originating from the pancreas. The patient died of a pulmonary embolism a few hours after the exploratory operation. A histological section of the tumour from the patient is shown in Fig. 1 together with the xenografted tumour from Passage 11.

*HX18.*—This tumour was derived from a suture-line recurrence in a 61-year-old patient following a hemicolectomy. The primary tumour removed 20 months previously was a poorly differentiated, mucin-secreting adenocarcinoma.

Both the xenografts were originally obtained by R. J. Pickard and HX18 was used in cell kinetic studies reported by Pickard, Cobb and Steel (1975).

*Cell suspensions.*—Tumours were removed under sterile conditions, rinsed in phosphate-buffered saline (PBS) and chopped into pieces of less than 1 mm diameter with crossed scalpels. It was necessary to adapt subsequent treatment to the requirements of different tumours. For tumour HX32, the pieces were incubated for 25 min at 37°C in 2 mg/ml collagenase (Worthington) in culture medium (Ham's F12 with 15% Special Bobby Calf Serum from Gibco-Biocult). After 2 washes in PBS the pieces were incubated for 5 min at 37°C in 0·05% trypsin (Bacto trypsin, Difco) and the supernatant decanted. The pieces were resuspended in Ham's F12 without serum, and incubation was continued for a further 3–5 min. The container was then shaken ×3 to dislodge cells from the pieces and, after the pieces had settled, the cell suspension was removed and serum added to a concentration of about 15% to stop the action of trypsin. The cells were centrifuged for 3 min at less than 1000 rev/min, resuspended in culture medium and then filtered through a 30 $\mu$m polyester mesh (Henry Simon, Stockport). After standing 15–20 min at 4°C (7 ml of medium in a 17 mm-diameter test tube) the top $\frac{2}{3}$ of the suspension was taken for use. Cells were examined in a haemocytometer under phase contrast and those that did not take up lissamine green and had an intact and smooth outline, were scored as viable.

For Tumour HX18, collagenase treatment was not required. Trypsinization at 37°C was continued for 20 min with a change to fresh trypsin at 10 min. The suspension was finally filtered through a 20 $\mu$m mesh.

Heavily irradiated (HR) cells were prepared from tumour cell suspensions exposed to 10,000 rad $\gamma$-rays from a $^{60}$Co source.

*Red cells.*—Blood was obtained by cardiac puncture from August rats and the buffy coat removed after centrifugation. The red blood cells were rinsed ×3 by resuspending in PBS and centrifuging, and finally made up to the original volume with culture medium. Aliquots not exceeding 10 ml were stored at 4°C in universal containers for periods up to 20 days. RBC used within 1 week were heated to 44°C for 1 h to destroy residual nucleated cells.

*Agar medium.*—A 5% agar solution was made by boiling powdered agar (Bacto agar, Difco) in double-distilled water for 10 min. After cooling to 44°C, 1 vol of 5% agar was mixed with 9 vols of culture medium at 44°C.

*Culture procedure.*—To set up a number of replicate tubes, 1 vol of RBC suspension diluted 1/4 plus 1 vol of HR tumour cells ($10^5$/ml) was added to a test tube containing 2 vols of tumour cell suspension. After warming to 37°C, 6 vols of 0·5% agar at 44°C was added and mixed. Aliquots of 1 ml were pipetted into individual tubes and immediately plunged into crushed ice to set.

FIG. 1.—Histological sections of the pancreatic tumour HX32. ×300. (a) Section of the original tumour removed from the patient at the time of operation. (b) Xenografted tumour at Passage 11. (c) Xenograft grown from cells maintained in culture for 2½ months.

FIG. 1(c)

The tubes were gassed for 5 sec using a mixture of 5% $O_2$ and 90% $N_2$ and 5% $CO_2$ delivered at 2 l/min *via* a Pasteur pipette, and the caps were tightened. Gassing was repeated 20–30 min later after the tubes had been warmed to 37°C in a waterbath. The capped tubes were not perfectly airtight and the gas concentration was maintained by incubating the tubes in (6 × 12 × 17 cm) transparent polystyrene boxes (Stewart Plastics) gassed with the same mixture *via* holes in opposite sides of the box and finally sealed with polythene adhesive tape. To check for gas leaks, an indicator tube containing 1 ml medium with phenol red indicator but without serum was placed in each box. The tubes were maintained in a vertical position and incubated at 37°C.

After 5 days, when red cells had lysed, 2 ml of medium was pipetted on top of the agar. At 12 and 20 days the medium was changed; the tubes being gassed on each occasion. The boxes were gassed twice weekly.

After 28 days the agar was decanted on to a slide, cut into 3 pieces and covered with a (25 × 50 mm) coverslip. Colonies (>50 cells) were counted using a magnification of ×40.

### RESULTS

### *Experiments on culture conditions*

Fig. 2 shows the results of measurements with Tumour HX32 designed to test the linearity of growth response over a range of cell concentrations and under various culture conditions. Groups of 5 replicate tubes were set up by the standard procedure with RBC and with or without HR cells. Tumour cells were added at a range of concentrations and the tubes were incubated with a gas phase containing 5% $O_2$ + 5% $CO_2$ + 90% $N_2$ with the exception of 2 additional groups of tubes in which the $O_2$ concentration was increased to 20%, the concentration in air. With 5% $O_2$ a plating efficiency (PE) of ~30% was obtained over the range 25–500 cells per tube, demonstrating linearity between colony number and the number of cells plated out. However, the addition of $10^4$ HR cells increased PE to 42%. Consequently, for survival experiments, HR cells were added as a standard

FIG. 2.—Plating efficiency plotted against number of tumour cells plated, using a gas phase containing 5 or 20% $O_2$. ○, 5% $O_2$ with $10^4$ HR cells; ×, 5% $O_2$ without HR cells; ∧, 20% $O_2$ without HR cells.

procedure to bring the total number of cells to $10^4$ per tube.

Colonies were also obtained in tubes gassed with 20% $O_2$ but the PE was only 20% and the colonies tended to be rather smaller. This result shows that the lower $O_2$ tension of 5% gave a useful increase in PE and slightly improved cell growth, but to a smaller extent than previously found for the Lewis lung tumour.

Rat RBC, previously shown to enhance the growth of mouse tumours, were similarly effective with tumour HX32, and results showing that RBCs gave a 3-fold increase in PE are given in Table I. The growth factor in RBC is released only after cell lysis, which occurs over a period of 5–7 days under our culture conditions, thus making the growth factor available at the critical time when colony growth is initiated. Blood from August rats was much superior to that from Wistar and Marshall rats, whose RBCs fail to lyse within the first week of culture. The addition of lysates instead of whole RBC was also found to be less effective, due to the instability of the growth factor after release from the RBC. Probably for the same reason, RBC added to the liquid phase instead of to the agar failed to give a good PE, suggesting that the growth factor was broken down before there was time for it to diffuse through the agar.

The results in Table I demonstrate the effect of two different RBC concentrations on PE, using RBC stored for up to 16 days. Each tube was seeded with 500 tumour cells without adding HR cells and RBC were obtained from 3 different batches of blood taken 2, 7 and 16 days previously. The 2- and 7-day RBCs, but not the 16-day RBCs, were heat-treated to destroy the nucleated cells. The results show no deterioration in the PE of blood stored for up to 16 days. In separate experiments with RBCs stored for 3 weeks or more, appreciable lysis was observed and such lysed RBCs were less effective in promoting cell growth.

The results obtained using a RBC dilution of 1/2 and 1/4 showed no difference between the 2 dilutions. The 1/2 dilution had previously been used for the growth of mouse tumour cells but on the basis of these results the 1/4 dilution has been adopted for the standard procedure.

There were indications, in preliminary experiments, that after 10–14 days in culture toxic substances were produced, possibly from the breakdown of released haemoglobin which at this stage was brown in colour. However, the addition of culture medium above the agar, in the standard procedure, dilutes the toxic products to a harmless level.

TABLE I.—*Effect of RBC on Plating Efficiency of HX*32

| Storage time (days) | Treatment | RBC dilution | PE (%) |
|---|---|---|---|
| 2 | Heated | 1/2 | 24·7 ± 1·6 |
| 2 | Heated | 1/4 | 20·1 ± 0·5 |
| 7 | Heated | 1/2 | 25·6 ± 0·7 |
| 7 | Heated | 1/4 | 26·6 ± 1·5 |
| 16 | Unheated | 1/2 | 28·4 ± 1·9 |
| 16 | Unheated | 1/4 | 26·0 ± 0·8 |
| No blood added | | — | 9·5 ± 0·9 |

TABLE II.—*Plating Efficiencies Obtained from Various Xenografted Tumours*

| Tumour type | Total examined | No. of tumours with PE >0·1% | PE (%) | Tumour line | Growth period weeks |
|---|---|---|---|---|---|
| Colorectal | 12 | 6 | 0·1 | | 5 |
| | | | 1·9 | | 5 |
| | | | 1·4 | | 5 |
| | | | 0·4 | | 4 |
| | | | 0·3 | HX18 | 4 |
| | | | 0·4 | | 4 |
| Oat-cell Ca | 3 | 2 | 0·3 | HX29 | 4 |
| | | | 3·0 | HX33 | 4 |
| Pancreatic | 2 | 2 | 30–50 | HX32 | 4 |
| | | | 3·0 | | 4 |
| *Melanoma | 1 | 1 | 8–12 | HX34 | 5 |
| *Uterine | 1 | 1 | 9·8 | HX35 | 3 |
| Teratoma | 1 | 0 | | | |

* Grown in culture before passaging in mice.

The culture technique has now been applied to the growth of a number of other xenografted tumours, and a second pancreatic tumour has given colonies with a PE of 3% (Table II). Colorectal tumours grow less well, and out of 12 tumours tested, only 6 gave colonies in agar, with PE of 0·1 to 1·9%. Colonies were also obtained from 2 of 3 oat-cell carcinomas of the lung. These tumours gave suspensions without the use of enzymes. However, the single cells were fragile and many lost the ability to exclude lissamine green within 2 h after the preparation of cell suspensions. The low PE obtained could therefore have been due to the high proportion of damaged or dying cells in the suspension.

Two other xenografted tumours, a uterine adenocarcinoma and a spindle-cell melanoma gave higher PE, of about 10%. Both of these tumours had been maintained in culture before implantation into immune-suppressed mice. The melanoma was derived from a biopsy specimen obtained in the clinic and grown in monolayer culture for 2 months before passaging in the mouse. In the xenograft, the characteristic morphology of the cells was retained and melanosomes were identified in the cytoplasm by electron microscopy.

The uterine tumour was obtained as a metastasis from a 74-year-old woman from whom an adenocarcinoma of the uterus had been removed 8 years previously. A cell suspension from the tumour removed at surgery was prepared and plated out by the standard method in agar. From a cell concentration of $10^4$ per tube, colonies were obtained with a PE of 12%. Some of the colonies were picked out and used to establish a monolayer culture which was subsequently passaged into immune-suppressed mice. The PE of cells from the xenograft was 9·8%, comparable to that of the cells taken directly from the original biopsy specimen.

Colonies have also been obtained from a number of other tumours obtained as biopsy specimens in the clinic and the results will be reported elsewhere.

In order to establish whether the xenografts had retained their human chromosome complement, chromosome preparations were made from cell suspensions prepared from the solid tumour in all the xenografts used in these studies. It was found that the cells had retained a human karyotype, with acrocentric and metacentric chromosomes. A minority of cells (less than 1%) possessed only the telocentric chromosomes typical of mouse cells, and these were taken to represent mouse-derived stromal cells. There was no evidence of hybridization between human and mouse cells. After 16 days in monolayer culture, cells of Tumours

HX18 and HX32 showed no mouse chromosomes in over 200 metaphase spreads counted. Cultures seeded from agar colonies were morphologically similar to those grown in monolayer culture directly from the mouse. Conclusive evidence that the cells growing in culture were tumour cells and representative of those in the xenograft was first obtained for the HX18 tumour. After 2 months growth in culture, $5 \times 10^5$ HX18 cells were injected s.c. into each of 20 mice, and in 5 of the mice tumours were produced at the site of injection. On microscopic examination these were indistinguishable from the original xenograft. A similar result was obtained from cultured HX32 cells implanted into mice. Fig. 1 shows a histological section of this tumour for comparison with the regularly passaged HX32 tumour and they can be seen to be closely similar.

*Application of the* in vitro *agar assay*

The assay technique has been applied to the measurement of cell survival in solid tumours treated with various cytotoxic agents. As an example, a radiation-survival curve for the HX32 tumour exposed to γ-rays is shown in Fig. 3. The tumours were irradiated *in situ* in the mouse and removed for assay 18 h later. A number of different cell dilutions were set up in agar, and from the colony count the surviving fraction was calculated by dividing the PE by that of the untreated controls. The points shown on the curve were obtained in 7 different experiments and they give an indication of the reproducibility of the technique.

FIG. 3.—Cell-survival curve for Tumour HX32 treated in the mouse with γ-rays.

FIG. 4.—Cell-survival curve for Tumour HX18 treated in the mouse with cyclophosphamide and assayed by the agar diffusion chamber technique in parallel with the *in vitro* assay. ○, *in vitro* assay; △, agar diffusion chamber assay.

Fig. 4 shows a cell-survival curve for the colonic tumour HX18 treated *in vivo* with cyclophosphamide. The drug was injected i.p. into tumour-bearing mice in single doses of up to 300 mg/kg, the maximum tolerated dose, and the tumours were removed 18 h later and cell suspensions prepared for assay. At the same time a parallel assay was carried out using the agar diffusion chamber method (Smith, Courtenay and Gordon, 1976). With this technique, tumour cells are suspended in agar in diffusion chambers which are implanted into the peritoneal cavity of previously irradiated mice. The chambers are incubated within the mouse for about 3 weeks, during which time nutrient substances in the peritoneal fluid are able to reach the tumour cells by diffusion. Cell survival values obtained by the 2 methods were comparable, and it was concluded that the more artificial growth conditions of the *in vitro* assay did not influence the recovery of the drug-treated cells.

### DISCUSSION AND CONCLUSIONS

The agar colony technique we have developed has been designed for the growth of relatively slowly proliferating tumour cells by using a replenishable liquid phase above the agar which permits continued growth throughout the 4 or more weeks necessary for the production of colonies of suitable size. The addition to the agar of rat RBC, which lyse and release a labile growth factor available to the growing cells, consistently improved the PE of cells from a number of different human tumour xenografts. The requirement for the growth factor may well be a characteristic of cells in primary culture, since RBC also enhance the growth of marrow, and also of Lewis lung and B16 mouse tumour cells taken directly from the animal.

Of the 20 different xenografted tumours studied, 12 gave rise to colonies in agar. However, considerable differences in PE were observed for different tumours. The majority of tumours tested were of colorectal origin, and half of these gave colonies in agar, but the PE was low (<2%). This could be because the proportion of clonogenic cells in the tumour was small, or because of some special metabolic requirement of these cells. It could also be associated with cellular damage sustained in the preparation of the cell suspension and the higher PE of ~10% obtained from a melanoma and a uterine tumour, which yielded cell suspensions without the use of enzymes, supports this possibility.

The pancreatic tumour HX32 which gave the highest PE (averaging 30%) is particularly suitable for experimental use and has been the subject of further radiation studies (Courtenay *et al.*, 1976; Smith, Courtenay and Steel, 1978). Some of the other tumours studied here have also been used to measure clonogenic cell survival after treatment with cytotoxic agents. The agreement between the results obtained in parallel experiments on the colonic tumour HX18 using agar diffusion chambers implanted in the mouse and the *in vitro* agar method has provided additional evidence of the validity of the *in vitro assay.*

The successful growth of colonies from a tumour taken directly from a patient has also demonstrated the application of the culture technique for the growth of cells taken directly from human biopsy material, and the method has now been used to grow colonies from a number of other tumours obtained in the clinic (in preparation).

We wish to thank Dr G. G. Steel and Professor L. F. Lamerton for their support and encouragement, Dr N. M. Blackett for helpful discussion, Mr D. M. Melville for expert technical assistance, Dr K. Nowak, Mr J. A. McKinna and Mr J. E. Gibbs for supplying the tumours and Dr Shirley Kauffman for the photographs.

### REFERENCES

BRADLEY, T. R., TELFER, P. A. & FRY, P. (1971) The Effect of Erythrocytes on Mouse Bone Marrow Colony Development *In vitro*. *Blood*, **38**, 353.

CASTRO, J. E. (1972) Human Tumours Grown in Mice. *Nature, New Biol.*, **239**, 83.

COBB, L. M. (1973) The Behaviour of Carcinoma of the Large Bowel in Man Following Transplantation into Immune-deprived Mice. *Br. J. Cancer*, **28**, 400.

COURTENAY, V. D. (1976) A Soft Agar Colony Assay for Lewis Lung Tumour and B16 Melanoma Taken Directly from the Mouse. *Br. J. Cancer*, **34**, 39.

COURTENAY, V. D., SMITH, I. E., PECKHAM, M. J. & STEEL, G. G. (1976) *In vitro* and *In vivo* Radiosensitivity of Human Tumour Cells. *Nature, Lond.*, **263**, 771.

PICKARD, R. G., COBB, L. M. & STEEL, G. G. (1975) The Growth Kinetics of Xenografts of Human Colorectal Tumours in Immune-deprived Mice. *Br. J. Cancer*, **31**, 36.

SANDERS, F. K. & BURFORD, B. O. (1964) Ascites Tumours from BHK 21 Cells Transformed *In vitro* by Polyoma Virus. *Nature, Lond.*, **201**, 786.

SMITH, I. E., COURTENAY, V. D. & GORDON, M. Y. (1976) A Colony Forming Assay for Human Tumour Xenografts using Agar in Diffusion Chambers. *Br. J. Cancer*, **34**, 476.

SMITH, I. E., COURTENAY, V. D. & STEEL, G. G. (1978) The *In vitro* Radiation Response of Cells from 4 Human Tumours Propagated in Immune-suppressed Mice. *Cancer Res.* (in press).